# Spatio-temporal dynamics of soil bacterial communities as a function of Amazon forest phenology

**DOI:** 10.1038/s41598-018-22380-z

**Published:** 2018-03-12

**Authors:** Erika Buscardo, József Geml, Steven K. Schmidt, Helena Freitas, Hillândia Brandão da Cunha, Laszlo Nagy

**Affiliations:** 1 Centre for Functional Ecology - Science for People & the Planet, University of Coimbra, Coimbra, Portugal; 2 Department of Plant Biology, University of Campinas, Campinas, Brazil; 3 Large-scale Biosphere-Atmosphere Programme, National Amazonian Research Institute, Manaus, Brazil; 4 Biodiversity Dynamics Research Group, Naturalis Biodiversity Center, Leiden, The Netherlands; 5 Faculty of Sciences, Leiden University, Leiden, The Netherlands; 6 Department of Ecology and Evolutionary Biology, University of Colorado, Boulder, USA

## Abstract

Most tropical evergreen rain forests are characterised by varying degrees of precipitation seasonality that influence plant phenology and litterfall dynamics. Soil microbes are sensitive to soil water:air ratio and to nutrient availability. We studied if within-year seasonality in precipitation and litterfall-derived nutrient input resulted in predictable seasonal variation in soil bacterial diversity/microbial functional groups in an Amazonian forest. We characterised the spatio-temporal dynamics of microbial communities from the plot to the stand scales and related them to precipitation seasonality and spatial variability in soil characteristics. Community composition and functional diversity showed high spatial heterogeneity and was related to variability in soil chemistry at the stand level. Large species turnover characterised plot level changes over time, reflecting precipitation seasonality-related changes in soil nutrient and moisture regimes. The abundance of decomposers was highest during the rainy season, characterised also by anaerobic saprophytes and N_2_-fixers adapted to fluctuating redox conditions. In contrast, Beijerinckiaceae, likely derived from the phyllosphere, were found at higher abundances when litter inputs and accumulation were highest. We showed that in a mildly seasonal rain forest, the composition of soil microbial communities appears to be following canopy phenology patterns and the two are interlinked and drive soil nutrient availability.

## Introduction

Studies of tropical forest phenology have shown correlations between patterns of precipitation seasonality, litterfall peaks and new leaf production, with ensuing patterns for gross primary production (photosynthesis)^1,2^. Such temporal pattern in phenology, more specifically peak periods of litterfall, must also affect soil/below-ground processes. In fact, the dynamics and composition of soil microbes in terrestrial ecosystems and the biogeochemical processes that they are associated with can vary greatly both in time^3^ and space^4^. Temporal changes in bacterial communities vary from prompt responses to environmental cues to seasonal within-year dynamics^5^. Seasonality in microbial communities is linked to the temporal variability of plant-derived resources^6^, which, in turn, is related to limiting climatic factors that constrain growth during the year. Along with temporal variability there is a spatially uneven distribution of plant-derived resources as a result of differences in plant species composition/spatial distribution, quality in plant tissue chemistry and their seasonal production that may differ among different coexisting species^7^. The complexity of spatio-temporal variation makes the study of seasonal changes in microbial communities difficult and requires contemporaneous studies that also consider the magnitude of spatial soil heterogeneity^8^.

Variations in soil bacterial communities in extratropical natural ecosystems in response to seasonal changes in environmental variables have been reported from alpine tundra^9,10^ to temperate forests^11,12^. Across these different biomes, there have been similar taxonomic shifts observed between seasons, i.e. a higher relative abundance of Actinobacteria in winter and of Acidobacteria and certain Proteobacteria in summer^9–11^. These changes have been attributed to seasonal fluctuations in carbon substrate quality and availability associated with growing seasons in temperate ecosystems^5^ that are known to have short- and medium-term impacts on soil bacterial communities^13^.

The above ecosystems are characterised by distinct growing seasons, ranging from 3 months in alpine vegetation to 6–7 months in temperate forests, and they starkly contrast with lowland tropical evergreen rain forests (LTERF), where there is no such temperature limitation to plant growth. However, LTERF are characterised by varying degrees of precipitation seasonality (defined by the presence or absence of a period with negative hydrological balance – ‘dry *vs*. rainy’), which has been linked to phenological cycles and photosynthetic activity^14^, pulses in nutrient availability^15^ and to strong litterfall/fine root turnover patterns^16,17^. Nutrient pulses caused by environmental fluctuation (e.g. wetting and drying cycles) can result in concentrated release of readily soluble organic matter (OM), leachates from epiphytes, or from microbial cell lysis^15^. Periods of increased uptake of nutrients by plants may be related to fluctuations in soil moisture and pulses in nutrient availability, which in turn are associated with periodic crashes in soil microbial populations. Litterfall inputs and accumulation are generally highest during the dry season^2,16,18,19^. This contributes to large fluxes of dissolved OM early in the rainy season^18^ and stimulate microbial nutrient immobilization, thus reducing leaching during the rainy period^15,20^. The rainy season is also characterised by increased fine root biomass^17,19^, with potential but still unexplored links in rhizodeposition dynamics, and increased rates of soil respiration^19–21^ and decomposition of litter^16,22,23^ when compared with the dry season. Microbial communities are influenced directly by precipitation dynamics that cause temporal fluctuations in soil pore water to air ratio^24^ and moisture^25^, and indirectly through temporal variations in litterfall/fine root dynamics.

While some accounts exist on seasonal variability in biogeochemical processes in tropical forests driven by soil microbiota, e.g., decomposition/mineralization^26,27^, nitrification and denitrification^28–30^, and N_2_-fixation^31,32^, knowledge of the seasonality of soil bacterial communities is currently lacking. The work we report contributes to filling this knowledge gap.

**Figure 1:**
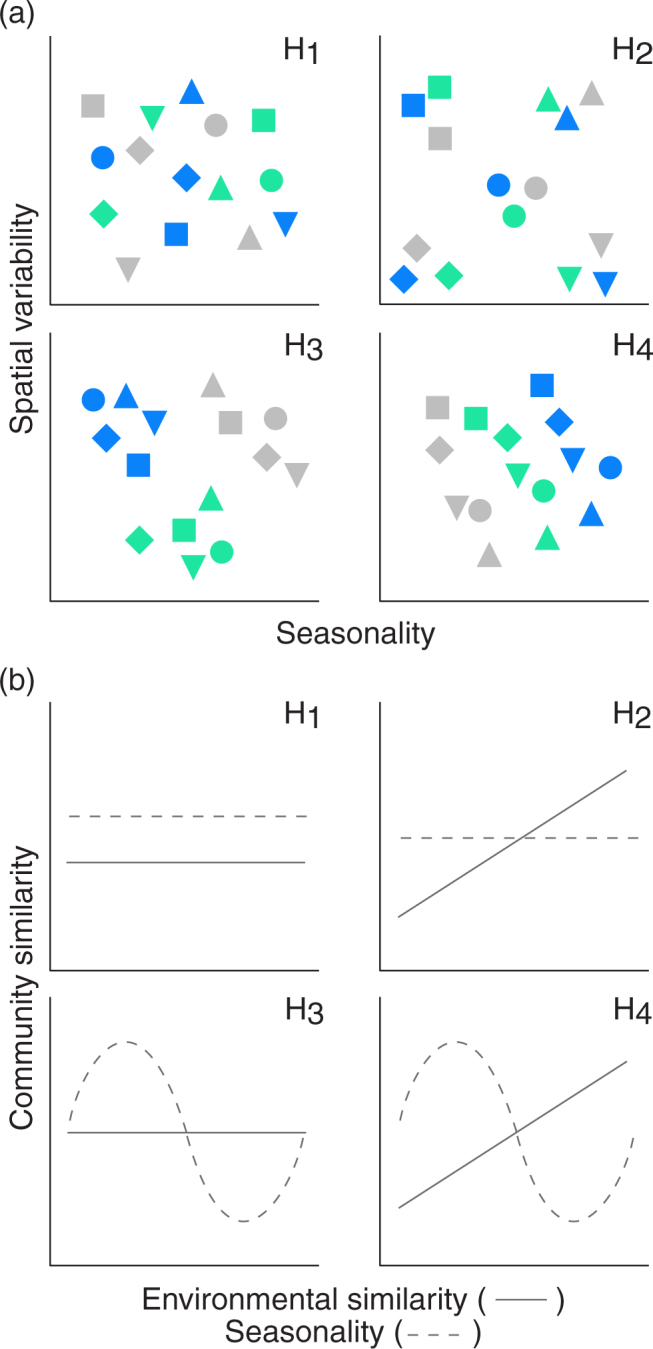
Four alternative hypotheses on the potential influence of spatial and temporal (i.e. seasonality) variability on soil bacterial community composition in a LTERF on *terra firme* in Amazonia, Brazil (modified after Martiny, *et al*.^77^). H_1_, bacterial communities are randomly distributed both in space and time (i.e. they are not influenced by resource spatial variability and by seasonality patterns); H_2_, bacterial community composition is influenced by resource spatial variability, but not by seasonality patterns; H_3_, bacterial community composition is influenced by seasonality patterns, but not by resource spatial variability; H_4_, bacterial community composition reflects both spatial and temporal conditions. For readability five sample points of the 15 in the present study are shown. Different colours represent samples collected during the rainy season (green), at the transition between rainy and dry season (blue), and during the dry season (grey).

We characterised spatio-temporal changes in bacterial community composition in a LTERF (*terra firme*), by amplicon sequencing the 16 S rDNA region. To determine the influence of spatial pattern (i.e. soil chemistry) over a scale that ranged from <1 m to >1 km and within-year temporal variability (i.e. seasonality in precipitation and litterfall-derived nutrient input: Time 1, wet season; Time 2, transition; Time 3, dry season) on soil bacterial communities/functional groups (defined here as process mediators such as decomposers, N_2_-fixers, nitrifiers, denitrifiers, methanogens, methane oxidizers, sulphate- and Fe(III)- reducing bacteria) we addressed four alternative hypotheses (Fig. 1). In an ecosystem that lacks a restriction of the growing season by low temperature, (H_1_) bacterial community composition is not affected by spatial variability in soil characteristics or by precipitation and litterfall/fine root related seasonality patterns; (H_2_) bacterial community composition is affected by spatial variability of resources but not by seasonality; (H_3_) bacterial community composition is affected by precipitation and litterfall/fine root related seasonality patterns (i.e. as the dry season is characterised by higher litterfall inputs and lower fine root biomass than the rainy season, dry season bacterial communities differ significantly from rainy season communities) and this community change overrides any spatial variability; and (H_4_) bacterial community composition reflects both spatial and temporal heterogeneity, i.e. bacterial community patterns are shaped by both high spatial heterogeneity and seasonality factors.

## Results

### Bacterial community characterization assessment

**Figure 2:**
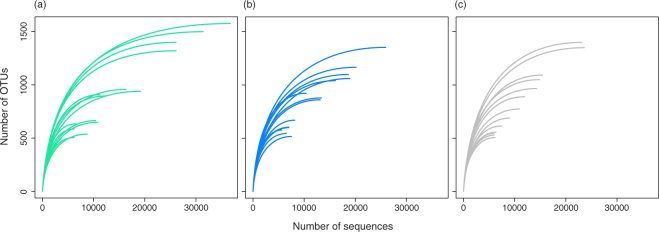
Rarefaction curves representing the relationship between the number of sequences (obtained by Ion Torrent sequencing of bacterial 16 S rDNA amplicon libraries generated from DNA extracted from soil samples collected in a LTERF during the rainy season - Time 1, at the transition between rainy and dry season - Time 2, and during the dry season - Time 3), and the number of operational taxonomic units (OTUs), assigned at 97% sequence similarity. Time 1, green; Time 2, blue; Time 3, grey.

Sequence read numbers after quality control and removal of all OTUs that had less than six occurrences across all samples averaged 14,075 (range 5,555–36,690) per sample and resulted in a total 3,714 OTUs over the three sampling dates. Species richness (SR), Shannon Index (H’) and the abundance-based coverage estimator (ACE) per plot averaged 723 ± 24 (range, 498–995), 5.3 ± 0.05 (range, 4.8–5.8) and 937 ± 50 (range, 522–1582), respectively. Phylogenetic diversity (PD) per plot averaged 163 ± 3 (range, 130–199). No significant differences were found for SR, H’ and PD, for any paired comparisons between sampling dates (data not shown). Rarefaction curves tended to saturation for almost every sample indicating that OTU’s were representative of the total bacterial community (Fig. 2).

Of all sequences, a total of 82.9% were classifiable to a taxonomic level. Proteobacteria were represented by an average relative abundance of 32.2% across samples, followed by Acidobacteria (21.5%), Actinobacteria (16%) and Firmicutes (13.5%). All other phyla except for Chloroflexi (2.9%), were represented on average by <1% of the total abundance. Within Proteobacteria, Alpha-Proteobacteria dominated with 65% of the relative abundances, followed by Beta-Proteobacteria (23%), Delta-Proteobacteria (7%) and Gamma-Proteobacteria (5%).

### Spatio-temporal changes/pattern in bacterial diversity

**Figure 3:**
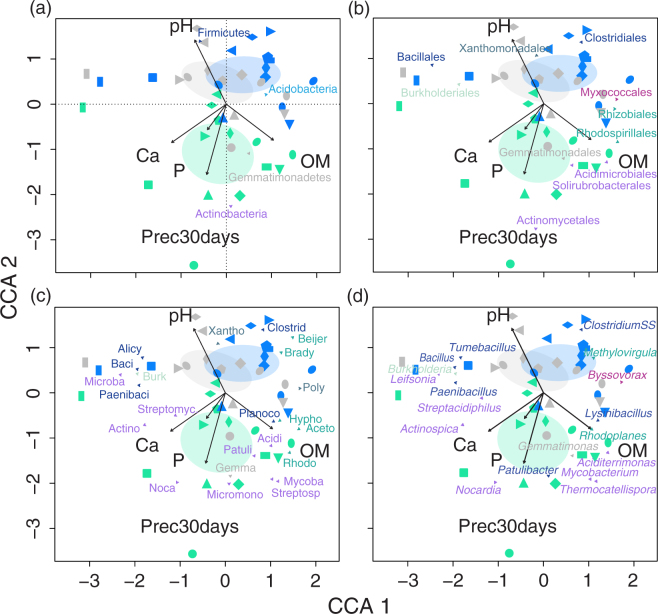
Canonical correspondence analysis (CCA) ordination plots based on relative abundances of rarefied taxonomic data of soil bacterial communities in response to seasonal dynamics in a LTERF forest. Total inertia, 3.53; Constrained 0.58; Eigenvalues CCA 1, 0.18; CCA 2, 0.13. Identical symbols in different colours refer to the individuals plots at the three sampling time. Time 1, green (rainy season); Time 2, blue (transition into dry season); Time 3, grey (dry season). Significant environmental variables were included in the ordination following a sequential ANOVA significance assessment of each single term. Ellipses denote 95% confidence intervals using standard error of the weighted average plot scores at each sampling time. Vectors representing bacterial taxa at phylum and lower taxonomic levels were fit into the ordination by using the ‘vegan’ envfit function and their significance assessed under 999 permutations. Because of the high number of taxa, vectors are distributed over four identical ordination plots (**a**), phylum; (**b**), order; (**c**), family, (**d**), genus; identical colour to identify taxa within a phylum). Prec30days, precipitation in the 30 days preceding sampling; OM, soil organic matter; P, phosphate; Ca, calcium; Acidi, Acidimicrobineae *Incertae Sedis*; Actino, Actinospicaceae; Microba, Microbacteriaceae; Micromo, Micromonosporaceae; Mycoba, Mycobacteriaceae; Noca, Nocardiaceae; Streptomy, Streptomycetaceae; Streptosp, Streptosporangiaceae; Patuli, Patulibacteraceae; Alicy, Alicyclobacillaceae; Baci, Bacillaceae 1; Paenibaci, Paenibacillaceae 1; Planoco, Planococcaceae; Clostrid, Clostridiaceae 1; Gemma, Gemmatimonadaceae; Beijer, Beijerinckiaceae; Brady, Bradyrhizobiaceae; Hypho, Hyphomicrobiaceae; Aceto, Acetobacteraceae; Rhodo, Rhodospirillaceae; Burk, Burkholderiaceae; Poly, Polyangiaceae; Xantho, Xanthomonadaceae.

ADONIS and PERMANOVA analyses carried out on UniFrac distance matrices calculated on both presence/absence and relative abundance data, showed significant effects of both seasonality and site on bacterial community composition (Table 1). Relationships between UniFrac distances and explanatory variables were observed for pH (F Model, 2.59; R^2^, 0.06; p ≤ 0.001), soil organic matter (OM, F Model, 2.22; R^2^, 0.05; p ≤ 0.01), PO_4_^3−^ (F Model, 1.56; R^2^, 0.03; p ≤ 0.05), Ca^2+^ (F Model, 2.36; R^2^, 0.05; p ≤ 0.001) and precipitation in the 30-day period preceding sampling (F Model, 1.95; R^2^, 0.04; p ≤ 0.01). The same variables were selected as significant in the canonical correspondence analyses (CCA) made using relative abundance of OTU data (Fig. 3; for statistical significance of individual variables see Table S1). The majority of taxa represented from order to genus level, belonged to three phyla: Actinobacteria, Alpha-Proteobacteria and Firmicutes (see Table S2 for significance of correlation). Relative abundances of different taxa belonging to Actinobacteria were positively related with the precipitation in the 30 days prior soil sampling and to soil Ca^2+^ and PO_4_^3−^ concentration. The abundance of Firmicutes was positively related to pH, whereas Hyphomicrobiaceae, Acetobacteraceae and Rhodospirillaceae (Alpha-Proteobacteria) were related to OM, and Beijerinckiaceae and Bradyrhizobiaceae increased in abundance in samples collected at Times 2 and 3 relative to Time 1.

**Figure 4:**
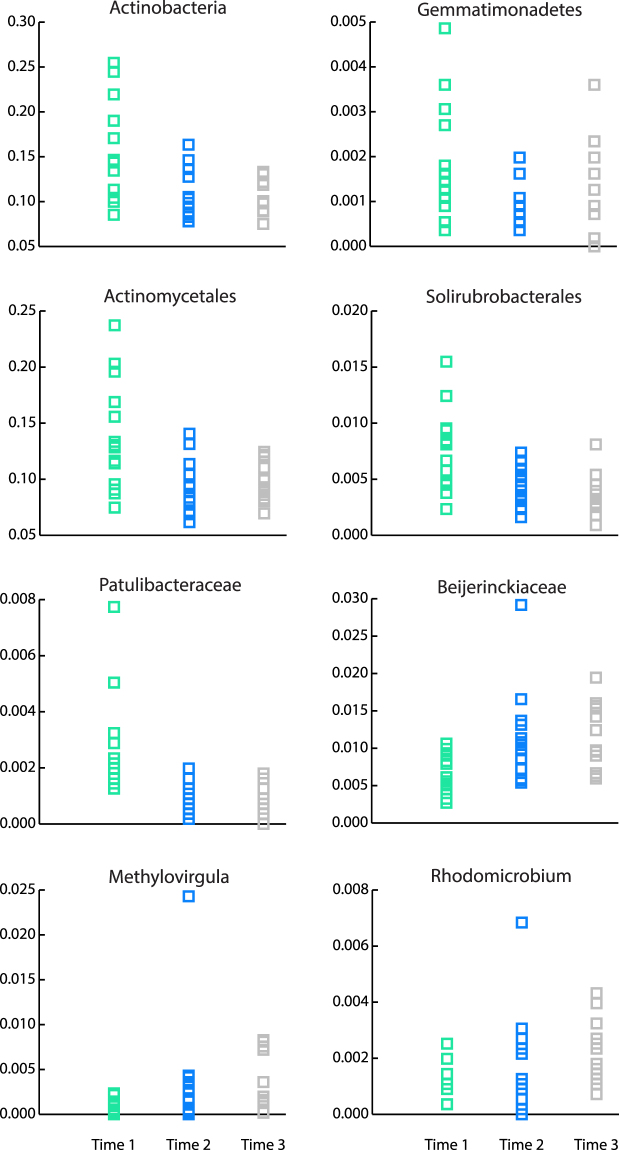
Relative abundances of bacterial taxa that significantly changed in response to seasonality in precipitation and litterfall in a LTERF. Time 1, green (rainy season); Time 2, blue (transition into dry season); Time 3, grey (dry season). For Gemmatimonadetes identical abundance values were found at all taxonomic levels and therefore only the phylum level figure is shown; for Patulibacteraceae and Patulibacter identical values are represented by Patulibacteraceae.

Significant shifts in bacterial community composition at all taxonomic levels were observed among the three sampling dates after correcting significances for multiple comparisons with the FDR method (Table S3). At the phylum level Actinobacteria decreased significantly in relative abundance from Time 1 to both Times 2 and 3, while Gemmatimonadetes decreased from Time 1 to Time 2. At a finer taxonomic resolution, the relative abundances of Actinomycetales, Solirubrobacterales and Gemmatimonadales were higher at Time 1 than Times 2 and 3. Patulibacteraceae showed higher relative abundances at Time 1 than Times 2 and 3 and Beijerinckiaceae higher relative abundances at Time 3 (and an increasing trend at Time 2) than Time 1. Three genera, Patulibacter, Methylovirgula and Rhodomicrobium exhibited statistically significant differences in pair-wise comparisons among seasons (Fig. 4; Table S3).

The bacterial community at each sampling date was characterised by distinct groups of indicator species (Table S4) - Time 1 (twenty-one indicators): Firmicutes (8), Proteobacteria (5) and Actinomycetales (4); Time 2 (twenty-one indicators): Proteobacteria (9), Bacteroidetes (3) and Firmicutes (3); and Time 3 (9 indicators): Acidobacteria (3). Times 1 and 2, and Times 1 and 3 shared 9 and 7 OTUs, respectively, mainly Actinobacteria, while Times 2 and 3 had twenty-four common OTUs, thirteen of which belonged to Proteobacteria.

### Spatio-temporal turnover: ß-diversity in space *vs*. time

Spatial turnover, calculated as species dissimilarity in plot-wise comparisons, was responsible for most of ß-diversity at the taxonomic level at all sampling dates (Time 1, ß_sim_ = 0.47, ß_sne_ = 0.08; Time 2, ß_sim_ = 0.48, ß_sne_ = 0.06; Time 3, ß_sim_ = 0.46, ß_sne_ = 0.08). At the phylogenetic level, there was a higher relative contribution of nestedness to ß _sor_, and values of ß-diversity were much lower than at taxonomic level (Time 1, ß_sim_ = 0.21, ß_sne_ = 0.07; Time 2, ß_sim_ = 0.22, ß_sne_ = 0.05; Time 3, ß_sim_ = 0.20, ß_sne_ = 0.06).

**Figure 5:**
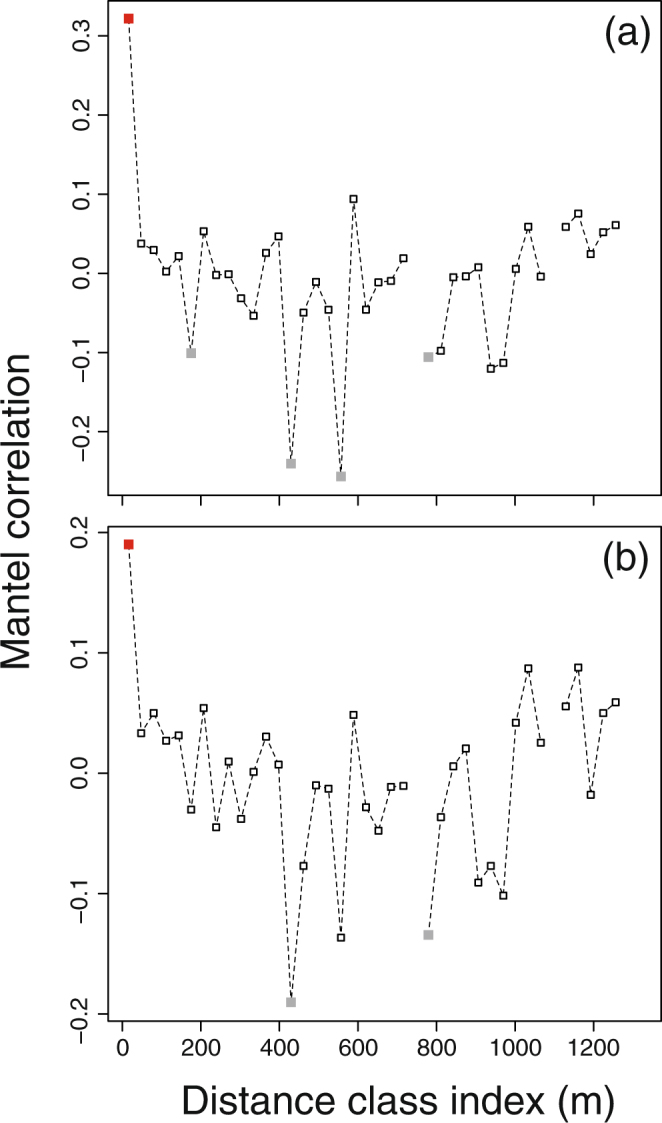
Mantel correlograms showing the correlations between soil bacterial community composition (ß_sor_) at different distance classes at taxonomic (**a**) and phylogenetic (**b**) levels. Filled squares indicate a significant Mantel r –value (red, positive; grey, negative).

Mantel correlograms calculated on all samples (i.e. ß_sor_ *vs*. geographical distance) showed that the composition of bacterial samples was positively autocorrelated at the plot level on both taxonomic (r = 0.32; corrected p-value < 0.001) and phylogenetic data (r = 0.19; corrected p-value < 0.001), indicating that samples collected at different sampling times at the same plot were not spatially independent (i.e. our sampling design allowed valid temporal comparisons), while the minimum distance between plots was greater than the scale of sample autocorrelation, suggesting that our plots represented spatially independent samples (Fig. 5).

Temporal changes in bacterial community composition at the plot level were strongly related to species turnover for all paired comparisons of sampling dates (ß_sim_ = 0.43, ß_sne_ = 0.05, for Time 1 and Time 2; ß_sim_ = 0.42, ß_sne_ = 0.05 between Time 2 and Time 3; ß_sim_ = 0.40, ß_sne_ = 0.07 between Time 1 and Time 3). The relative magnitude of variability at the stand level (Whittaker’s β_space_, 4.02) was, on average, 2.2 times higher than seasonal variability (β_time_, 1.85) at the plot level (Fig. S1).

### Soil analyses

Soil pH, mineral N, K^+^, exchangeable acidity (hydrogen, H^+^ plus aluminium, Al^3+^) and cation exchange capacity (CEC) were significantly different among sampling dates (Table 2). Values of pH increased successively from Time 1 to Time 3. Concentrations of both NH_4_^+^ and NO^3−^ were significantly less at Time 2 than at Time 1 and higher at Time 3 than Time 2 or Time 1. K^+^ was significantly higher at Time 3 than Times 1 and 2 while exchangeable acidity and CEC had an opposite pattern and both decreased from Time 1 to Time 3 and from Time 2 to Time 3. Fine root biomass was significantly higher at Time 1 when compared with Times 2 and 3. For correlation among variables of soil chemical properties see Table S5.

**Table 1:** Overview of the effects of seasonality and site (space/sampling point) on the bacterial community obtained with the ADONIS and PERMANOVA analyses carried out on UniFrac distance matrixes calculated on rarefied data of both presence/absence and relative abundance.

	Degrees of freedom	Sum of squares	Mean squares	F model	R^2^	Pr (>F)	
**Presence/Absence**							
ADONIS	Seasonality	2	0.062	0.031	1.69	0.07	9.999e-05***
Nested.npmanova	Site	14	0.417	—	0.97	—	9.999e-05***
**Relative abundance**							
ADONIS	Seasonality	2	0.094	0.047	2.25	0.07	9.999e-05***
Nested.npmanova	Site	14	0.671	—	1.02	—	9.999e-05***

**Table 2:** Seasonal dynamics of soil chemical composition and root biomass in a lowland evergreen rain forest in central Amazonia. Time 1 (rainy season); Time 2 (transition into dry season); Time 3 (dry season). Values are averaged data (n = 15 for all except mineral N and root biomass with n = 20) followed by standard error. Letters in superscript indicate statistically significant differences between sampling times as determined by paired t-test and Wilcoxon signed-rank test.

	Time 1	Time 2	Time 3
pH	3.75 ± 0.06^a^	3.87 ± 0.05^b^	4.27 ± 0.06^c^
NH_4_^+^ (μg g^−1^)	26.6 ± 1.6^a^	17.1 ± 1.2^b^	51.8 ± 1.2^c^
NO_3_^−^ (μg g^−1^)	2.98 ± 0.3^a^	1.93 ± 0.2^b^	5.07 ± 0.7^c^
PO_4_^3−^ (μg g^−1^)	11.21 ± 0.38	10.86 ± 0.43	10.46 ± 0.4
K^+^ (mmolc kg^−1^)	1.36 ± 0.07^a^	1.39 ± 0.09^a^	1.58 ± 0.11^b^
Ca^2+^ (mmolc kg^−1^)	4.31 ± 0.96	3.92 ± 0.79	3.71 ± 0.7
Mg^2+^ (mmolc kg^−1^)	1.72 ± 0.16	1.93 ± 0.19	1.68 ± 0.09
H + Al^2+^ (mmolc kg^−1^)	205.4 ± 15.4^a^	193.5 ± 14.9^a^	155.9 ± 12.2^b^
Organic matter (%)	7.52 ± 0.49	7.68 ± 0.45	7.12 ± 0.38
CEC (mmolc kg^−1^)	213.7 ± 15.8^a^	200.8 ± 15.1^a^	162.8 ± 12.3^b^
Fine roots (Mg ha^−1^)	7.03 ± 0.73^a^	4.5 ± 0.34^b^	4.56 ± 0.27^b^
Coarse roots (Mg ha^−1^)	2.75 ± 1.27^a^	3.29 ± 1.4^a^	6.52 ± 1.77^b^

## Discussion

This is the first study that provides insights into the spatio-temporal dynamics of soil bacterial communities in a tropical lowland evergreen rain forest (LTERF). Bacterial community composition and functional diversity were, on the one hand, related to soil resource patterns, and on the other, to seasonality-related changes in soil nutrients and moisture regimes. Community assemblages were largely structured by spatial turnover. Patterns of ß-diversity at the plot level were overwhelmingly attributable to species replacement - either in space (within a sampling date) or in time across three sampling dates, of the same plot. The relative magnitude of variability, calculated by Whittaker´s beta was twice as large at the stand level than seasonal variability at the plot level.

Spatial heterogeneity in soil chemical properties is determined by abiotic and biotic controls. Their relative importance in LTERF is expected to shift from abiotic to biotic control from regional to landscape and local spatial scales^33^ with plants exerting strong biotic controls at the plot scale^34^. Soil microbial community structure seems to follow identical pattern, being influenced by broad-scale environmental drivers at the regional/landscape scale, e.g. soil pH^35^, soil moisture^36^ and temperature^37^, while local-scale relationships are more likely to be influenced by plant-soil interactions^38,39^. While disentangling the effects of plant species on bacterial composition was outside the scope of the present study, we did observe that bacterial communities collected at different dates were spatially structured and positively autocorrelated at the plot level. This confirms the existence at the local scale of spatial horizontal patterns likely linked to the effects of plant species. Different species affect soils both directly through the chemistry of their litter^7,40,41^ and indirectly through the effect on detritivores, and litter Ca appears to be an important general agent in these processes^42^. Bacterial community composition in our study was significantly related to Ca^2+^, PO_4_^3^^−^^3^ and soil organic matter (OM) that did not follow a seasonal pattern and which are likely to reflect plant biotic control on spatial soil variability, and by pH, which did show a seasonal pattern with increasing values from the rainy to the dry season. In the light of these results we can reject both hypothesis H_1_ (bacterial communities are not affected by spatial variability of resources and by seasonality patterns) and hypothesis H_3_ (bacterial community composition is affected by seasonality patterns and this change overrides any spatial variability) and at this point, partially accept hypotheses H_2_ and H_4_ whereby in an ecosystem that lacks a restriction of the growing season by climate, bacterial community composition is influenced by spatial variability of resources but not by seasonality (H_2_), and that bacterial community composition reflects both spatial and temporal heterogeneity (H_4_).

The rainy season was characterized by a relative high abundance of Actinobacteria (i.e. Actinomycetales and Solirubrobacteriales) and a group of indicator species mainly represented by Actomycetales (e.g. Streptomycetaceae) and Firmicutes (e.g. *Paenibacillus* and *Clostridium*), that include major decomposers of complex polymers, anaerobic saprophytes and anaerobic N_2_-fixers adapted to fluctuating redox conditions^43^. The temporal changes in the abundance of Actinobacteria could have been partially driven by interactions between seasonal changes in nutrient and water availability. We found Actinobacteria to be correlated with precipitation in the month preceding sampling. This finding is in agreement with a study conducted in agricultural and temperate grassland ecosystems^8^ where the authors have suggested that the pattern could be attributed either to the taxa preference for moisture or, alternatively to a presumed late seasonal peak in plant litter and root exudates that could favour Actinobacteria over more oligotrophic taxa. With the start of the rainy season abundant daily rainfall promotes the movement of readily decomposable dissolved organic C from the litter layer^21,44^, driving high annual rates of OM decomposition^16,22^. Since different bacterial taxa process OM at different rates, even under similar abiotic conditions, shifts in OM resources could drive changes in microbial community composition^45^. For example, Actinobacteria have been shown to be able to survive when resources are limited or in the presence of complex, recalcitrant OM^46^. Actinomycetales are major decomposers of complex polymers such as lignocellulose and chitin^47,48^ and their higher abundance toward the end of the rainy season seems to be linked to changes in soil moisture and nutrient availability that promote optimal conditions for the decomposition of recalcitrant C.

Seasonal changes in soil moisture are linked to fluctuations in the availability of soil N, which appears to be related to the delicate balance between the processes of immobilization and mineralisation^49^. Luizão, *et al*.^20^ have observed in Amazonia that rewetting of the soil after a dry period induced net N immobilization, whereas net N mineralisation during dry periods resulted in mineral N accumulation in the soil. Net N mineralisation and nitrification in the study area were also higher in the dry season compared with those in the rainy season, with highest N mineralisation at the peak of the dry season^20,50^. In our study, we detected a higher mineral N concentration at the end of the dry season, when compared with values at the other sampling dates.

In tropical rain forest soils, microbial communities under fluctuating moisture regime appear to be characterised primarily by specialist taxa that are able to maintain several processes under fluctuating conditions^24,43^. For example, DeAngelis, *et al*.^43^ have shown that taxa which are significantly more active under fluctuating rather than static oxic conditions are mainly represented by Clostridia, Flavobacteria and Actonomycetales. This could have also been the case at our site at Time 1, characterized by highest abundance of Actinomycetales and by a high number of indicator species belonging to Firmicutes. These include taxa involved in the anaerobic metabolism of complex carbon, such as aerobic/facultative anaerobic Paenibacillaceae, as well as obligate anaerobic taxa involved in N_2_-fixation^51^.

Conversely, soil samples at the transition between rainy and dry seasons (Time 2) and at the end of the dry season (Time 3) were characterized by higher values of pH and K^+^ and a significantly higher relative abundance of Beijerinckiaceae belonging to Rhizobiales, when compared with their values in the rainy season. Times 2 and 3 shared also a relatively high number of indicator species belonging to Alpha-Proteobacteria. While species belonging to Beijerinckiaceae are known to be free-living N_2_-fixers^52^, we suspect that their higher relative abundance at Times 2 and 3 could not be related to potential higher rates of N_2_ fixation at these times of the year. Some studies conducted in LTERF have indeed observed higher rates of free-living N_2_-fixation in the rainy season when compared to the dry one, and attributed them to the co-occurrence during this period of C availability and favourable leaf litter N:P ratios^31,32^. To investigate the effects of OM content on soil microbial community structure, Nemergut, *et al*.^45^ manipulated litter inputs *in situ*. Interestingly, they found that the double-litter plots were characterized by a higher abundance of Rhizobiales than the control plots and suggested that the observed shifts could have been related to changes in N_2_-fixation. However, we suggest that another plausible explanation for the high abundance in their double-litter plots, and at our study site, both at Times 1 and 3, could perhaps be attributed to an increased input of these bacteria from the phyllosphere^53^ through increased litterfall^2,16^. Recent studies conducted in lowland tropical rain forests during the dry season^54^ and at the transition between dry and rainy season^55^, have shown that the most dominant taxa in the phyllosphere were taxa of Beijerinckiaceae and within them genera known to associate closely with plant hosts, including N_2_-fixing and methylotrophic taxa^54^. In the present study, the significant higher abundance of Beijerinckiaceae at the end of the dry season when compared with the rainy season, and almost significant at the transition between rainy and dry season would support the possibility of a higher abundance of bacteria in the soil linked to the phyllosphere when litter inputs and accumulation are at the highest^56^.

Since our results indicated that bacterial community composition is influenced by both spatial and temporal variability of resources we are able to reject hypothesis H_2_ in relation to seasonality, and accept hypothesis H_4_, whereby both spatial and temporal heterogeneity contribute significantly in shaping the composition of bacterial communities.

In conclusion, soil bacterial community composition and functional diversity were related to soil resource patterns and to seasonality-related changes in soil nutrient and moisture regimes in an Amazonian LTERF. The highest relative abundance of decomposers of complex polymers were found during the rainy season that was also characterised by indicators species that included anaerobic saprophytes and anaerobic N_2_-fixers adapted to fluctuating redox conditions. Beijerinckiaceae likely derived from the phyllosphere, were found at higher abundances when litter inputs and accumulation were highest. The composition of bacterial communities showed high spatial heterogeneity due to spatial variability in soil chemistry at the stand level and temporal changes that reflected seasonality at the plot level. To corroborate whether some of the reported changes are associated with large inputs of phyllosphere bacteria during the litterfall peak or are instead due to internal soil community dynamics requires further work to establish a functional link between canopy phenology and corresponding temporal patterns in microbial community composition and functioning.

## Materials and Methods

### Study site

The study was carried out in a 10-km^2^ forest reserve (Adolpho Ducke Forest Reserve, RFAD), 26 km NE of Manaus (03°00′00″ − 03° 08′00″ S; 59°52′40″ − 59° 58′00″W), Brazil, in 2013. The RFAD has a dissected undulating topography at 40–140 m a.s.l. and it is associated with different soil types: oxisols on plateaux, ultisols on slopes and hydromorphic spodosols in the valleys^57^. The plateau soils are well-drained and are not affected by waterlogging or flooding during the rainy season. The dominant vegetation type is lowland evergreen rain forest^58^. For further details see Oliveira Freitas, *et al*.^59^.

**Figure 6:**
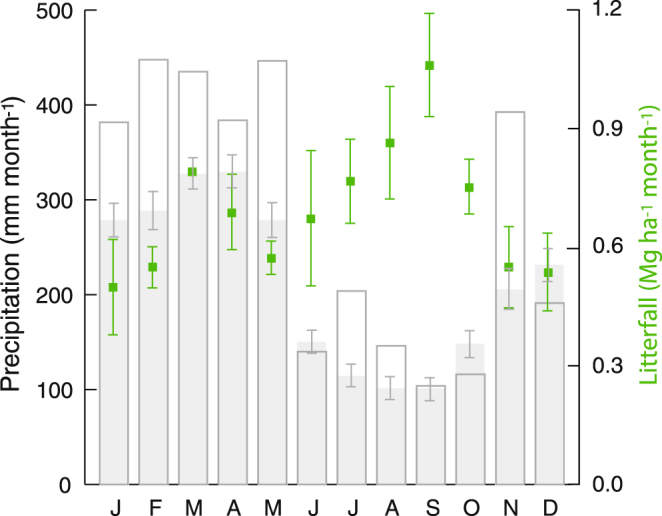
Average annual monthly rainfall and standard errors (SE) measured at the meteorological station of the study site during the period 1966–2014 (filled) and for the year 2013 (unfilled). In green the mean monthly litterfall inputs (±SE) from a LTERF forest in proximity of our study site over a three year period 1979–1982^16^.

The mean annual temperature is 26 °C and mean annual precipitation is 2,550 mm/year (range 1840–3385 mm/year for the period 1966–2014, measured at the Reserve’s meteorological station). Periods with monthly precipitation <100 m usually occur between September and November. Although the year of the study, 2013, was much wetter than the average and had no month with a perceived negative hydrological balance, the overall precipitation pattern did not deviate from the long-term mean (Fig. 6).

Litterfall patterns were established based on 3 years of data 1979–1982^16^. The study was designed to capture soil microbial community dynamics during a year, (1) in the wet season, (2) in a transition period during the displacement of the Inter-tropical Transition Zone to the north and the subsequent diminution of rainfall, and (3) at the end of the ‘dry season’.

### Data Accessibility

This Targeted Locus Study project has been deposited at DDBJ/EMBL/GenBank under the accession KBUV00000000. The version described in this paper is the first version, KBUV01000000.

### Sampling

Twenty points were chosen randomly along 1.5 km section in a plateau area of a 6-km trail that runs east to west in the RFAD. The minimum distance between any two points was set to be of 40 m for full sampling design see^60^. Soil was sampled on 13 April 2013 (wet season, Time 1), on 28 July (transition into dry season, Time 2) and on 6 November 2013 (dry season, Time 3). At every point, one 1 m × 1 m plot was marked permanently and in every plot three sub-samples were collected to a depth of 5 cm, after the removal of litter, using a cylindrical metal corer of 3 cm of diameter. Each sub-sample was sampled for DNA extraction by using a sterile needle and pooled by plot at each sampling in a 2.5-ml Eppendorf vial. The samples were maintained at −20 °C until molecular analyses. The remaining soil was pooled per plot for soil analyses and for the determination of fine root biomass at each sampling date.

### Molecular analyses

Molecular analyses for the characterisation of bacterial communities were carried out using a total of 45 samples (i.e. 15 of the original 20 replicates were chosen for molecular analyses). DNA extractions were carried out for each sample using a PowerSoil DNA Isolation Kit (MOBIO) with 250 mg of soil. The primers 27 F and 338 R^61,62^ were used for amplification of bacterial 16 S rDNA. The forward primer was linked through a barcode adaptor with the Ion-Xpress barcode and the Ion adaptor ‘A’ sequence, while the reverse primer was linked with the P1 sequence (Ion Torrent adaptors, primers and barcodes used in the present study are available in Table S6 of Supporting information). For details on PCR settings see Buscardo, *et al*.^60^. Negative controls consisting of nuclease free water instead of DNA were made for every barcode and subjected to PCR. Amplicon libraries were quantified using a Qubit 3.0 Fluorometer using a Qubit HS dsDNA Assay Kit (Life Technology). Equimolar amounts of each library were pooled (24 barcode per chip), purified with the Agencourt AMPure XP Kit (Beckam Coulter) and diluted to 100 pM. The final solution for emulsion PCR was obtained by diluting 3 μl of the solution 100 pM in 22 μl of nuclease free water according to the Ion PGM Template OT2 400 Kit manual for further details see^60^. Amplicon libraries were sequenced using two Ion 318 Chips by an Ion Torrent Personal Genome Machine (PGM; Life Technologies) at the Institute of Biological Sciences, Federal University of Parà, Brazil.

### Sequence processing

The primers were removed and poor-quality ends were trimmed off based on 0.02 error probability limit in Geneious Pro 5.6.1 (BioMatters, New Zealand). Sequences were then filtered using USEARCH v.8.0^63^ as described in Buscardo, *et al*.^60^. After excluding singletons and discarding OTUs that were also present in any of the five negative samples, the data set contained 588,168 sequences in 7,888 OTUs. Since the reliability of OTUs with a low number of sequences may also be questioned and given the high total number of sequences per sample (between 195,000 and 370,000), we choose to follow the conservative approach suggested by^64^ and removed all OTUs that had less than six occurrences across all samples. For the final analyses the total number of sequences was thus reduced to 576,867 (3,714 OTUs). Sequences were assigned to taxonomic groups by RDP Naïve Bayesian Classifier Version 2.10^65^.

### Soil analyses

Ammonium (NH_4_^+^), nitrate (NO^3−^) and soil moisture were quantified in the Soil Laboratory of the National Research Institute of Amazonia (INPA), Manaus, Brazil for each soil sample (a total of 60 samples), within 48 h after collection for details see^60^. The remaining soil was air dried and pH, total soil organic matter (OM), extractable phosphorous (PO_4_^3−^), potassium (K^+^), calcium (Ca^2+^), magnesium (Mg^2+^), and exchangeable acidity (hydrogen, H^+^ plus aluminium, Al^3+^), were determined in the Laboratory of Soil Analysis, Escola Superior Agraria Luiz de Queiroz, University of São Paulo (ESALQ/USP; Piracicaba, Brazil), according to van Raij, *et al*.^66^. Fine (≤2 mm) and coarse roots were separated in each soil sample and their biomass determined by oven drying at 65 °C dry to constant mass.

### Data analyses

All analyses were conducted on data rarefied to 5,555 sequences (corresponding to the smallest number of sequences encountered in a sample). All analyses based on phylogenetic information were based on a phylogenetic tree built with an approximate maximum-likelihood approach with FastTree v.2.1^67^, built on a code edited with the –DUSE_DOUBLE parameter. The phylogeny was midpoint-rooted, using the Archaeopteryx interface^68^. The generalized UniFrac procedure^69^ implemented in the R package ‘GUniFrac’ v.1, was employed to compute UniFrac distances^70^. Rarefaction curves, α-diversity (SR), Shannon index (H’) and the abundance-based coverage estimator (ACE) were computed in the ‘vegan’ R package v.2.3–25^71^. Phylogenetic diversity was calculated as Faith’s phylogenetic diversity (PD) using the R package ‘picante’ v.1.6–2^72^. Taxonomic and phylogenetic ß-diversity were computed R package ‘betapart’ v.1.3^73^ to characterize spatial turnover (ß_sim_) and nestedness (ß_sne_) at each sampling date. Differences in diversity indices among community at different sampling dates were tested using pair-wise differences with the t-test.

To determine the relative magnitude of spatial and temporal variability in OTU richness, we calculated ß-diversity by using Whittaker’s orginal formula (β = γ/α), where γ is the total number of OTUs at each of the sampling ocassion (total of 15 plots for space and of three samplings per plot for time) and α is the number of OTUs per plot at a given time. We did this (a) for spatial variability (β_space_) at the stand level (full extent of the sampling transect) and (b) for temporal variability (β_time_) at the individual plot level.

Spatial autocorrelation between bacterial community composition (ß_sor_) at the sample level (within plot at different sampling dates and between plots) and geographical distances were assessed with a Mantel correlogram in the ‘vegan’ R package, using the Pearson’s correlation method with 9999 permutations. Distance classes were chosen based on the sampling distances within our sampling design. Significance values across distance classes were corrected for multiple comparisons using the progressive Holm method.

Temporal changes in taxonomic community composition were assessed on presence/absence data, by computing dissimilarities (‘betapart’ v.1.3 R package) for each sampled plot, between different sampling dates, and considering the overall change as well as the single components (i.e. ß_sim_ and ß_sne_) over time.

Spatial and temporal differences in phylogenetic community composition considering repeated-measures were assessed on rarefied presence/absence and relative abundance data with two different analyses, ADONIS, with sampling date nested in site to evaluate sampling date effect (‘vegan’ R package v.2.3–2), and nested PERMANOVA ‘BiodiversityR’ R package^74^; to evaluate the site effect, both using 10,000 permutations. ADONIS was also employed to quantify the relationship between UniFrac distances calculated on rarefied relative abundance data and soil explanatory variables. The GUniFrac distance matrix used in ADONIS was made by setting alpha to 0.5 to provide the best overall power^69^.

Canonical correspondence analyses (CCA) were carried out on relative abundance taxonomic data to visualize differences in OTU-based community composition. Only significant variables were included in the ordination following a sequential ANOVA significance assessment of each single term. Vectors representing bacterial taxa at phylum and lower taxonomic levels were fit into the ordination by using the ‘vegan’ ‘envfit’ function (only relative abundances of the first 300 most abundant OTUs were considered) and their significance assessed under 999 permutations.

Differences between sampling dates, in relative abundances of the first 300 most abundant OTUs (corresponding to 77% of the total abundance) were computed on data at phylum and at lower taxonomic levels and evaluated with the Wilcoxon signed-rank test. Significances were corrected for multiple comparisons using the false discovery rate (FDR) method^75^.

An indicator species analysis was carried out using ‘indicspecies’ v.1.7–6 in R^76^.

Differences in soil parameters at different sampling dates were evaluated with paired t-test or with the Wilcoxon signed-rank test when normality assumptions were not satisfied. Correlations between soil parameters were assessed using the Pearson’s correlation method.

## Electronic supplementary material

Supplementary Information
