# Supplementary material for: Spatio-temporal dynamics of soil bacterial communities as a function of Amazon forest phenology
Source: Sci Rep. 2018 Mar 12;8:4382. doi: 10.1038/s41598-018-22380-z (PMC5847513; doi:10.1038/s41598-018-22380-z)
Supplement: Supplementary file 1 — Supplementary Information [file 41598_2018_22380_MOESM1_ESM.pdf]

Spatio-temporal dynamics of soil bacterial communities in function of Amazon forest phenology

Erika Buscardo, József Geml, Steven K. Schmidt, Helena Freitas, Hillândia Brandão da Cunha, Laszlo Nagy

SUPPLEMENTARY INFORMATION (SI)

SI FIGURES LEGENDS

Figure S1. Correlation between ß-diversity calculated with the Whittaker's orginal formula ($\beta = \gamma/\alpha$) at the stand level, (β_{space}) and at the plot level (β_{time}).

SI TABLE CAPTIONS

Table S1. Significant soil variables included in a canonical correspondence analysis (CCAs) carried out on rarefied relative abundance of taxonomic data to visualize differences in OTU-based bacterial community composition. The variables were included in the ordination following a sequential ANOVA significance assessment of each single term.

Table S2. Correlation between taxa at phylum and lower taxonomic levels (as relative abundance of the first 300 most abundant OTUs) and ordination axis of a CCA performed on relative abundance of soil bacterial communities in a lowland evergreen rainforest in Amazonia. Correlations were obtained with the envfit function in the vegan R package under 999 permutations.

Table S3. Differences between relative abundances of bacterial taxa in soils collected a lowland Amazonian rain forest in response to seasonal dynamics. Changes were tested on rarefied relative abundances of the first 300 most abundant OTUs (corresponding to 75% of the total abundance). Differences were computed on data at phylum level and at lower taxonomic levels (i.e., genus, family, order and class depending on the taxonomic resolution available) and evaluated with the Wilcoxon signed-rank test. Significances were corrected for multiple comparisons using the false discovery rate (FDR) method. +, increased relative abundance; -, decreased relative abundance. Time 1, rainy season; Time 2, transition between rainy and dry season; Time 3, dry season.

Table S4. Overview of the results obtained with an indicator species analysis for bacterial communities in response to seasonal dynamics in a tropical lowland rain forest. Time 1, rainy season; Time 2, transition between rainy and dry season; Time 3, dry season.

Table S5. Correlations among soil chemical variables of samples collected in a lowland tropical rain forest.

Table S6. Overview of modified 27F and 338R oligonucleotide primers used to prepare amplicon libraries for Ion Torrent sequencing by PCR amplification of the bacterial V1-V2 hypervariable regions of 16S rRNA genes in DNA samples.

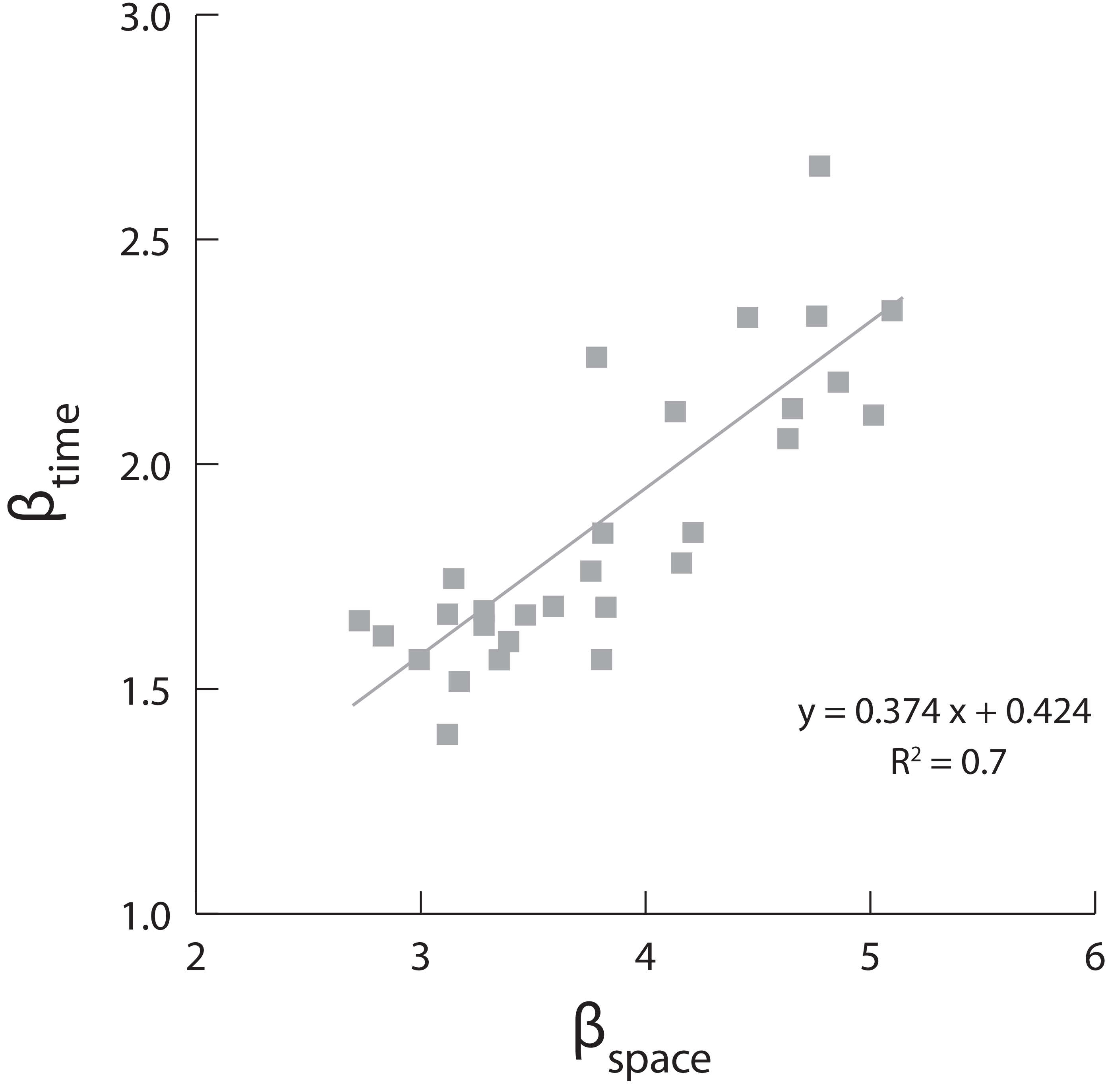

Figure S1.

Table S1.

<- cca(Seasonality3732RelAbun.rff~ PrecPrevMonth+pH+MO+P+Ca, data=Meta)
> ord
Call: cca(formula = Seasonality3732RelAbun.rff ~ PrecPrevMonth + pH +
MO + P + Ca, data = Meta)

Inertia	Proportion	Rank	
Total	3.5319	1	
Constrained	0.5766	0.1633	5
Unconstrained	2.9553	0.8367	35

Inertia is mean squared contingency coefficient
67 species (variables) deleted due to missingness

Eigenvalues for constrained axes:

CCA1	CCA2	CCA3	CCA4	CCA5
0.17946	0.1304	0.10832	0.08602	0.07242

Eigenvalues for unconstrained axes:

CA1	CA2	CA3	CA4	CA5	CA6	CA7	CA8
0.2748	0.20108	0.18566	0.15175	0.13021	0.12183	0.1186	0.1069

(Showed only 8 of all 35 unconstrained eigenvalues)

> anova(ord, by="term")
Permutation test for cca under reduced model
Terms added sequentially (first to last)
Permutation: free
Number of permutations: 999

Model:
cca(formula = Seasonality3732RelAbun.rff ~ PrecPrevMonth + pH + MO + P + Ca, data = Meta)

	Df	ChiSquare	F	Pr(>F)	
PrecPrevMonth	1	0.1233	1.4603	0.011	*
pH	1	0.11938	1.4139	0.015	*
MO	1	0.10609	1.2564	0.077	.
P	1	0.10623	1.2581	0.071	.
Ca	1	0.1216	1.4402	0.012	*
Residual	35	2.95528			

Signif. codes: 0 '***' 0.001 '**' 0.01 '*' 0.05 '.' 0.1 ' ' 1

Table S2.

	CCA1	CCA2	r2	Pr(>r)	
**PHYLUM**					
Acidobacteria	0.97177	0.23594	0.1325	0.068	.
Actinobacteria	0.04415	-0.99903	0.7995	0.001	***
Firmicutes	-0.38555	0.92269	0.3621	0.001	***
Gemmatimonadetes	0.41675	-0.90902	0.2298	0.008	**
**ORDER**					
Acidimicrobiales	0.73128	-0.68208	0.3655	0.001	***
Actinomycetales	-0.06422	-0.99794	0.7718	0.001	***
Solirubrobacterales	0.33788	-0.94119	0.1704	0.026	*
Bacillales	-0.94484	0.32752	0.6867	0.001	***
Clostridiales	0.52168	0.85314	0.2589	0.003	**
Gemmatimonadales	0.41675	-0.90902	0.2298	0.009	**
Rhizobiales	0.99724	-0.07422	0.2511	0.011	*
Rhodospirillales	0.88728	-0.46124	0.3428	0.001	***
Burkholderiales	-0.97517	0.22144	0.375	0.001	***
Myxococcales	0.99825	0.05921	0.265	0.003	**
**FAMILY**					
AcidimicrobineaeIncertaeSedis	0.73128	-0.68208	0.3655	0.002	**
Actinospicaceae	-0.92893	-0.37026	0.3579	0.001	***
Microbacteriaceae	-0.98554	0.16942	0.5343	0.001	***
Micromonosporaceae	0.04083	-0.99917	0.3926	0.001	***
Mycobacteriaceae	0.51144	-0.85932	0.501	0.001	***
Nocardiaceae	-0.47399	-0.88053	0.4891	0.001	***
Streptomycetaceae	-0.91078	-0.41289	0.1448	0.062	.
Streptosporangiaceae	0.47572	-0.8796	0.4559	0.001	***
Patulibacteraceae	0.05971	-0.99822	0.3299	0.002	**
Alicyclobacillaceae	-0.92028	0.39125	0.3842	0.002	**
Bacillaceae1	-0.96575	0.25946	0.3936	0.001	***
Paenibacillaceae1	-0.99618	0.08728	0.3554	0.002	**
Planococcaceae	0.89104	-0.45393	0.1794	0.023	*
Clostridiaceae1	0.51338	0.85816	0.2579	0.005	**
Gemmatimonadaceae	0.41675	-0.90902	0.2298	0.013	*
Beijerinckiaceae	0.77183	0.63583	0.3607	0.001	***
Bradyrhizobiaceae	0.81079	0.58534	0.2267	0.01	**
Hyphomicrobiaceae	0.85969	-0.51082	0.1511	0.048	*
Acetobacteraceae	0.89687	-0.44228	0.3191	0.002	**
Rhodospirillaceae	0.65364	-0.75681	0.2337	0.014	*
Burkholderiaceae	-0.97728	0.21197	0.3843	0.001	***
Polyangiaceae	0.99825	0.05921	0.265	0.003	**
Enterobacteriaceae	0.45719	0.88937	0.0721	0.182	
**GENUS**					
Aciditerrimonas	0.73128	-0.68208	0.3655	0.001	***
Actinospica	-0.92893	-0.37026	0.3579	0.003	**
Leifsonia	-0.98554	0.16942	0.5343	0.001	***
Mycobacterium	0.51144	-0.85932	0.501	0.001	***

Nocardia	-0.47399	-0.88053	0.4891	0.001	***
Streptacidiphilus	-0.99617	-0.08744	0.1833	0.028	*
Streptomyces	-0.85294	-0.52201	0.0931	0.17	
Thermocatellispora	0.47572	-0.8796	0.4559	0.001	***
Patulibacter	0.05971	-0.99822	0.3299	0.001	***
Tumebacillus	-0.92028	0.39125	0.3842	0.002	**
Bacillus	-0.96146	0.27494	0.4037	0.001	***
Paenibacillus	-0.99356	0.11329	0.3708	0.002	**
Lysinibacillus	0.89104	-0.45393	0.1794	0.019	*
ClostridiumSS	0.51496	0.85721	0.2589	0.005	**
Gemmatimonas	0.41675	-0.90902	0.2298	0.01	**
Methylovirgula	0.82149	0.57022	0.1788	0.037	*
Rhodomicrobium	0.61461	0.78883	0.1154	0.104	
Rhodoplanes	0.7963	-0.6049	0.1754	0.029	*
Burkholderia	-0.97728	0.21197	0.3843	0.001	***
Byssovorax	0.99091	0.13456	0.2946	0.004	**
Erwinia	0.45719	0.88937	0.0721	0.186	

Signif. codes: 0 '***' 0.001 '**' 0.01 '*' 0.05 '.' 0.1 ' ' 1

Permutation: free
Number of permutations: 999

Table S3.

	Time 1	Time 2	Time 3	corrected p-vlaue 1 vs 2	1 vs 3	2 vs 3	SIGN 1 vs 2	SIGN 1 vs 3	SIGN 2 vs 3
**PHYLUM**									
Acidobacteria	0.180069	0.165899	0.19694	0.3872	0.6978	0.3042	-	+	+
Actinobacteria	0.159316	0.105941	0.110711	0.0250	0.0471	0.6878	-	-	+
Bacteroidetes	0.001556	0.002186	0.001828	0.1548	0.6978	0.6878	+	+	-
Cand. Saccharibacteria	0.002777	0.008127	0.004265	0.4130	0.4813	0.7335	+	+	-
Chloroflexi	0.009515	0.005889	0.015883	0.6811	0.6978	0.3042	-	+	+
Cyanobacteria	0.002829	0.002687	0.002451	0.8001	0.6978	0.7335	-	-	-
Firmicutes	0.074296	0.15479	0.080787	0.1548	0.6978	0.3042	+	+	-
Gemmatimonadetes	0.001852	0.000759	0.001274	0.0250	0.6578	0.3042	-	-	+
Planctomycetes	0.000746	0.00045	0.000762	0.3930	0.6978	0.3042	-	+	+
Proteobacteria	0.226218	0.267635	0.278931	0.6761	0.4250	0.6878	+	+	+
**ORDER**									
Acidimicrobiales	0.003382	0.002623	0.002049	0.8903	0.12665	0.38025	-	-	-
Actinomycetales	0.137997	0.092015	0.096753	0.04372	0.08025	0.49971	-	-	+
Solirubrobacterales	0.007445	0.004359	0.00367	0.05735	0.02816	0.49971	-	-	-
Sphingobacteriales	0.001556	0.002186	0.001828	0.18579	0.77267	0.49971	+	+	-
Ktedonobacterales	0.009515	0.005889	0.015883	0.8903	0.77267	0.38025	-	+	+
Bacillales	0.055754	0.04683	0.054725	0.8903	0.90654	0.49971	-	-	+
Clostridiales	0.017667	0.106699	0.024912	0.10838	0.9612	0.2154	+	+	-
Gemmatimonadales	0.001852	0.000759	0.001274	0.04372	0.65775	0.38025	-	-	+
Planctomycetales	0.000746	0.00045	0.000762	0.50529	0.90654	0.38025	-	+	+
Rhizobiales	0.067558	0.070387	0.068988	0.8903	0.90654	0.9051	+	+	-
Rhodospirillales	0.048823	0.044542	0.051859	0.67069	0.90654	0.49971	-	+	+
Burkholderiales	0.039038	0.064446	0.070456	0.8903	0.621	0.49971	+	+	+
Myxococcales	0.008062	0.008139	0.007408	0.8903	0.90654	0.49971	+	-	-
Enterobacteriales	0	0.009104	2.77E-05	0.19865	0.71829	0.49971	+	+	-
Xanthomonadales	0.000862	0.002225	0.006647	0.8903	0.15184	0.2154	+	+	+

FAMILY

AcidimicrobineaeIncertae	0.003382	0.002623	0.002049	0.79917	0.15198	0.633	-	-	-
Actinospicaceae	0.001337	0.000939	0.000526	0.3432	0.77354	0.86448	-	-	-
Microbacteriaceae	0.001594	0.001839	0.002299	0.78205	0.9806	0.86448	+	+	+
Micromonosporaceae	0.01642	0.010582	0.011313	0.06687	0.13035	0.82042	-	-	+
Mycobacteriaceae	0.043629	0.030654	0.030368	0.06894	0.13035	0.8895	-	-	-
Nocardiaceae	0.016292	0.001569	0.004404	0.3432	0.94368	0.633	-	-	+
Pseudonocardiaceae	0.000604	0.000733	0.000457	0.84696	0.9806	0.86448	+	-	-
Streptomycetaceae	0.005709	0.002353	0.006453	0.2035	0.92906	0.501	-	+	+
Streptosporangiaceae	0.001517	0.00063	0.000845	0.18561	0.501	0.86448	-	-	+
Conexibacteraceae	0.004706	0.003446	0.002742	0.57633	0.30956	0.81625	-	-	-
Patulibacteraceae	0.002739	0.000913	0.000928	0.00152	0.00402	0.954	-	-	+
Chitinophagaceae	0.001556	0.002186	0.001828	0.2035	0.9272	0.82042	+	+	-
Ktedonobacteraceae	0.002572	0.001723	0.006107	0.85146	0.9806	0.86448	-	+	+
Alicyclobacillaceae	0.005928	0.012125	0.013017	0.70232	0.94368	0.82042	+	+	+
Bacillaceae 1	0.033226	0.013244	0.022918	0.2035	0.94368	0.633	-	-	+
Bacillaceae 2	0	0.007535	0.002285	0.2035	0.501	0.86448	+	+	-
Paenibacillaceae 1	0.004796	0.001826	0.004417	0.24738	0.9272	0.82042	-	-	+
Planococcaceae	0.000669	0.000591	0.00054	0.85146	0.94368	0.92432	-	-	-
Clostridiaceae 1	0.01705	0.105889	0.024372	0.13605	0.9806	0.4017	+	+	-
Gemmatimonadaceae	0.001852	0.000759	0.001274	0.06687	0.71755	0.633	-	-	+
Planctomycetaceae	0.000746	0.00045	0.000762	0.41612	0.94368	0.61035	-	+	+
Beijerinckiaceae	0.006468	0.010968	0.011535	0.06894	0.03918	0.82042	+	+	+
Bradyrhizobiaceae	0.007381	0.009104	0.007492	0.35644	0.9806	0.633	+	+	-
Hyphomicrobiaceae	0.041353	0.036788	0.037568	0.72157	0.94368	0.898	-	-	+
Acetobacteraceae	0.046355	0.042626	0.049685	0.72157	0.94368	0.82042	-	+	+
Rhodospirillaceae	0.001813	0.001479	0.001606	0.85146	0.94368	1	-	-	+
Burkholderiaceae	0.038781	0.061695	0.069376	0.8903	0.5943	0.81625	+	+	+
Polyangiaceae	0.008062	0.008139	0.007408	0.8903	0.94368	0.82042	+	-	-
Enterobacteriaceae	0	0.009104	2.77E-05	0.2035	0.77354	0.81625	+	+	-
Xanthomonadaceae	0.000862	0.002225	0.006647	0.84696	0.20245	0.4017	+	+	+

GENUS									
Aciditerrimonas	0.00338	0.00262	0.00205	0.81693	0.16211	0.47712	-	-	-
Actinospica	0.00134	0.00094	0.00053	0.39223	0.71396	0.83829	-	-	-
Leifsonia	0.00159	0.00184	0.0023	0.79791	0.94957	0.83829	+	+	+
Mycobacterium	0.04363	0.03065	0.03037	0.09192	0.11376	0.85065	-	-	-
Nocardia	0.01629	0.00157	0.0044	0.39223	0.82572	0.51493	-	-	+
Streptacidiphilus	0.00127	0.00046	0.0014	0.17324	0.71396	0.58338	-	+	+
Streptomyces	0.00444	0.00189	0.00505	0.39223	0.78924	0.37525	-	+	+
Thermocatellispora	0.00152	0.00063	0.00084	0.17324	0.48096	0.83829	-	-	+
Conexibacter	0.00471	0.00345	0.00274	0.5824	0.33019	0.6464	-	-	-
Patulibacter	0.00274	0.00091	0.00093	0.00162	0.00429	0.95195	-	-	+
Chitinophaga	0.00059	0.00051	0.00093	1	0.71396	0.70568	-	+	+
SaccharibacteriaGeneral	0.00278	0.00813	0.00427	0.54293	0.48096	0.83829	+	+	-
Ktedonobacter	0.00257	0.00172	0.00611	0.9144	0.94957	0.83829	-	+	+
Streptophyta	0.00283	0.00269	0.00245	0.9144	0.82572	0.83829	-	-	-
Tumebacillus	0.00593	0.01213	0.01302	0.68267	0.86775	0.79177	+	+	+
Bacillus	0.02581	0.01094	0.0199	0.21173	0.7912	0.47712	-	-	+
Cohnella	0.00099	0.00066	0.00064	0.86707	0.7325	0.70568	-	-	-
Paenibacillus	0.00381	0.00117	0.00378	0.15141	0.7325	0.47712	-	-	+
Lysinibacillus	0.00067	0.00059	0.00054	0.9144	0.86775	0.92021	-	-	-
ClostridiumSS	0.01553	0.10503	0.02171	0.06326	1	0.32032	+	+	-
Gemmatimonas	0.00185	0.00076	0.00127	0.06326	0.7016	0.47712	-	-	+
Aquisphaera	0.00075	0.00045	0.00076	0.4716	0.82572	0.43403	-	+	+
Methylovirgula	0.00069	0.00363	0.00348	0.09811	0.02501	0.83829	+	+	-
Bradyrhizobium	0.00409	0.00473	0.00345	0.68267	0.7912	0.6464	+	-	-
Rhodomicrobium	0.00103	0.00171	0.00228	0.54293	0.02501	0.43403	+	+	+
Pedomicrobium	0.00181	0.00126	0.00224	0.97961	0.71396	0.43403	-	+	+
Rhodoplanes	0.03851	0.03382	0.03304	0.6928	0.7325	1	-	-	-
Burkholderia	0.03878	0.06169	0.06938	0.96243	0.57629	0.6464	+	+	+
Byssovorax	0.0005	0.0008	0.00105	0.39223	0.33019	0.78976	+	+	+
Erwinia	0	0.0091	2.8E-05	0.25427	0.71396	0.6464	+	+	-
Dyella	0.00013	0.00198	0.00593	0.97961	0.48096	0.51493	+	+	+
Frateuria	0.00073	0.00024	0.00072	0.4716	0.71396	0.32032	-	-	+

Table S4.

Time 1 #sps. 21

	stat	p.value						
OTU263	0.635	0.01	**	Actinobacteria	Actinobacteria	Actinomycetales	Streptomycetaceae	
OTU158	0.522	0.047	*	Actinobacteria	Actinobacteria	Actinomycetales	Streptomycetaceae	*Streptomyces*
OTU420	0.739	0.003	**	Actinobacteria	Actinobacteria	Actinomycetales		
OTU2181	0.581	0.018	*	Actinobacteria	Actinobacteria	Actinomycetales		
OTU8510	0.495	0.047	*	Chloroflexi	Ktedonobacteria	Ktedonobacterales	Thermosporotrichaceae	*Thermosporothrix*
OTU973	0.606	0.022	*	Firmicutes	Bacilli	Bacillales	Paenibacillaceae 1	*Paenibacillus*
OTU4128	0.535	0.02	*	Firmicutes	Bacilli	Bacillales		
OTU5031	0.535	0.025	*	Firmicutes	Clostridia	Clostridiales	Lachnospiraceae	*Clostridium* XlVa
OTU994	0.585	0.028	*	Firmicutes	Clostridia	Clostridiales		
OTU2200	0.535	0.026	*	Firmicutes	Clostridia			
OTU1634	0.529	0.038	*	Firmicutes	Clostridia			
OTU864	0.676	0.008	**	Firmicutes				
OTU3632	0.542	0.043	*	Firmicutes				
OTU2616	0.535	0.029	*	Proteobacteria	Alphaproteobacteria	Rhizobiales		
OTU2730	0.594	0.007	**	Proteobacteria	Alphaproteobacteria			
OTU564	0.59	0.04	*	Proteobacteria	Alphaproteobacteria			
OTU4514	0.535	0.025	*	Proteobacteria	Deltaproteobacteria	Myxococcales	Polyangiaceae	*Byssovorax*
OTU1846	0.51	0.028	*	Proteobacteria	Gammaproteobacteria	Legionellales	Coxiellaceae	*Aquicella*
OTU1044	0.623	0.024	*	unclassified				
OTU832	0.613	0.022	*	unclassified				
OTU6317	0.535	0.03	*	unclassified				

Time 2 #sps. 21

	stat	p.value						
OTU1817	0.572	0.021	*	Acidobacteria	Gp1			
OTU1559	0.535	0.03	*	Acidobacteria	Gp13			
OTU7058	0.505	0.041	*	Actinobacteria	Actinobacteria	Actinomycetales		
OTU3396	0.598	0.008	**	Bacteroidetes	Cytophagales			
OTU69	0.568	0.013	*	Bacteroidetes	Sphingobacteriales	Sphingobacteriaceae	Mucilaginibacter	
OTU806	0.535	0.03	*	Bacteroidetes	Sphingobacteriia	Sphingobacteriales	Chitinophagaceae	*Chitinophaga*

OTU2325	0.628	0.042	*	cand. division WPS-1	WPS-1_genera_IS			
OTU32	0.821	0.003	**	Firmicutes	Clostridia	Clostridiales	Clostridiaceae 1	*Clostridium SS*
OTU363	0.633	0.024	*	Firmicutes	Clostridia	Clostridiales	Clostridiaceae 2	*Clostridium SS*
OTU8739	0.536	0.029	*	Firmicutes	Clostridia	Clostridiales	Clostridiaceae 2	*Clostridium SS*
OTU3729	0.5	0.041	*	Proteobacteria	Alphaproteobacteria	Alphaproteobacteria_IS		*Rhizomicrobium*
OTU2167	0.555	0.032	*	Proteobacteria	Alphaproteobacteria	Rhizobiales	Beijerinckiaceae	*Methylocapsa*
OTU2346	0.556	0.02	*	Proteobacteria	Alphaproteobacteria	Rhodospirillales	Acetobacteraceae	
OTU4268	0.535	0.025	*	Proteobacteria	Alphaproteobacteria	Rhodospirillales	Acetobacteraceae	
OTU2128	0.535	0.025	*	Proteobacteria	Alphaproteobacteria	Rhodospirillales	Rhodospirillaceae	
OTU2206	0.565	0.019	*	Proteobacteria	Alphaproteobacteria			
OTU4979	0.569	0.031	*	Proteobacteria	Deltaproteobacteria	Myxococcales	Cystobacteraceae	
OTU4035	0.62	0.047	*	Proteobacteria	Deltaproteobacteria	Myxococcales	Polyangiaceae	
OTU4297	0.632	0.015	*	Proteobacteria	Gammaproteobacteria			
OTU8312	0.535	0.03	*	unclassified				
OTU2488	0.502	0.038	*	unclassified				

Time 3 #sps. 18

	stat	p.value						
OTU4952	0.48	0.034	*	Acidobacteria	Gp1			Cand. *Koribacter*
OTU9281	0.48	0.034	*	Acidobacteria	Gp1			
OTU8576	0.48	0.027	*	Acidobacteria	Gp5			
OTU7416	0.493	0.047	*	Actinobacteria	Actinobacteria	Actinomycetales	Streptomycetaceae	*Streptomyces*
OTU6340	0.623	0.016	*	Actinobacteria	Actinobacteria	Actinomycetales		
OTU1985	0.521	0.013	*	Cand. Saccharibacteria	Saccharibacteria_genera_IS			
OTU180	0.48	0.026	*	Firmicutes	Negativicutes	Selenomonadales	Veillonellaceae	
OTU2584	0.554	0.023	*	Firmicutes				
OTU9011	0.48	0.028	*	Planctomycetes	Planctomycetia	Planctomycetales	Planctomycetaceae	
OTU7843	0.664	0.006	**	Proteobacteria	Betaproteobacteria	Burkholderiales	Burkholderiaceae	*Burkholderia*
OTU282	0.507	0.043	*	Proteobacteria	Betaproteobacteria	Burkholderiales	Burkholderiaceae	*Burkholderia*
OTU3662	0.593	0.008	**	unclassified				
OTU2045	0.59	0.005	**	unclassified				
OTU10846	0.555	0.005	**	unclassified				
OTU2066	0.525	0.013	*	unclassified				
OTU6234	0.48	0.028	*	unclassified				
OTU2696	0.48	0.028	*	unclassified				

OTU7639	0.48	0.023	*	unclassified				

Time 1+2 #sps. 9

	stat	p.value						
OTU4034	0.756	0.002	**	Acidobacteria	Gp1			
OTU3105	0.708	0.014	*	Actinobacteria	Actinobacteria	Actinomycetales	Micromonosporaceae	
OTU3143	0.738	0.013	*	Actinobacteria	Actinobacteria	Actinomycetales		
OTU6027	0.736	0.027	*	Actinobacteria	Actinobacteria	Actinomycetales		
OTU1715	0.642	0.038	*	Bacteroidetes	Sphingobacteriia	Sphingobacteriales	Chitinophagaceae	
OTU4987	0.723	0.019	*	Proteobacteria	Alphaproteobacteria	Rhizobiales	Bradyrhizobiaceae	*Bradyrhizobium*
OTU4853	0.627	0.022	*	Proteobacteria	Deltaproteobacteria	Myxococcales	Polyangiaceae	*Byssovorax*
OTU1173	0.722	0.003	**	unclassified				
OTU2318	0.719	0.01	**	unclassified				

Time 1+3 #sps. 7

	stat	p.value						
OTU135	0.817	0.024	*	Actinobacteria	Actinobacteria	Actinomycetales	Streptomycetaceae	*Streptomyces*
OTU3369	0.724	0.041	*	Actinobacteria	Actinobacteria	Actinomycetales	Streptomycetaceae	*Kitasatospora*
OTU7202	0.577	0.029	*	Actinobacteria	Actinobacteria	Actinomycetales	Streptomycetaceae	*Streptomyces*
OTU199	0.784	0.007	**	Actinobacteria	Actinobacteria	Actinomycetales		
OTU5173	0.753	0.014	*	Actinobacteria	Actinobacteria	Actinomycetales		
OTU562	0.599	0.046	*	Actinobacteria	Actinobacteria	Actinomycetales		
OTU6933	0.657	0.01	**	Firmicutes	Bacilli	Bacillales	Bacillaceae 1	*Bacillus*

Time 2+3 #sps. 24

	stat	p.value						
OTU651	0.805	0.011	*	Acidobacteria	Gp1			*Granulicella*
OTU5425	0.654	0.026	*	Acidobacteria	Gp2			
OTU264	0.932	0.001	***	Acidobacteria	Gp3			
OTU4694	0.782	0.013	*	Acidobacteria	Gp3			
OTU1781	0.749	0.045	*	Acidobacteria	Gp3			
OTU5637	0.795	0.006	**	Actinobacteria	Actinobacteria	Actinomycetales	Mycobacteriaceae	*Mycobacterium*
OTU1100	0.743	0.02	*	Bacteroidetes	Sphingobacteriia	Sphingobacteriales	Chitinophagaceae	*Segetibacter*
OTU495	0.709	0.045	*	Bacteroidetes	Sphingobacteriia	Sphingobacteriales	Chitinophagaceae	*Chitinophaga*
OTU4472	0.672	0.022	*	Chloroflexi	Ktedonobacteria	Ktedonobacterales	Ktedonobacteraceae	*Ktedonobacter*

OTU1153	0.756	0.036	*	Proteobacteria	Alphaproteobacteria	Rhizobiales		
OTU8589	0.623	0.024	*	Proteobacteria	Alphaproteobacteria	Rhizobiales		
OTU868	0.793	0.025	*	Proteobacteria	Alphaproteobacteria	Rhodospirillales	Acetobacteraceae	
OTU1378	0.694	0.007	**	Proteobacteria	Alphaproteobacteria	Rhodospirillales	Acetobacteraceae	*Granulibacter*
OTU2031	0.682	0.007	**	Proteobacteria	Alphaproteobacteria	Rhodospirillales	Acetobacteraceae	
OTU6980	0.649	0.019	*	Proteobacteria	Alphaproteobacteria			
OTU692	0.888	0.002	**	Proteobacteria	Betaproteobacteria	Burkholderiales	Burkholderiaceae	*Burkholderia*
OTU86	0.84	0.004	**	Proteobacteria	Betaproteobacteria	Burkholderiales	Burkholderiaceae	*Burkholderia*
OTU2259	0.609	0.032	*	Proteobacteria	Betaproteobacteria	Burkholderiales	Burkholderiaceae	*Burkholderia*
OTU1642	0.609	0.034	*	Proteobacteria	Deltaproteobacteria	Myxococcales		
OTU1167	0.667	0.015	*	Proteobacteria	Deltaproteobacteria			
OTU2162	0.772	0.007	**	Proteobacteria	Gammaproteobacteria	Xanthomonadales	Sinobacteraceae	*Steroidobacter*
OTU1773	0.799	0.012	*	Proteobacteria				
OTU980	0.705	0.02	*	unclassified				
OTU5457	0.577	0.043	*	unclassified				

Signif. codes: 0 ‘***’ 0.001 ‘**’ 0.01 ‘*’ 0.05 ‘.’ 0.1 ‘ ’ 1

Table S5.

	NH_4^+	NO_3^-	pH	MO	PO_4^{3-}	K^+	Ca^{2+}	Mg^2	H^+ plus Al^{3+}	CTC
NH_4^+	1									
NO_3^-	0.61***	1								
pH	0.57***	0.35*	1							
OM	0.03	-0.24	-0.23	1						
PO_4^{3-}	-0.09	0.12	-0.2	0.48***	1					
K^+	0.31*	0.12	0.39**	0.12	0.16	1				
Ca^{2+}	0.04	0.21	-0.04	0.21	0.34*	-0.18	1			
Mg^{2+}	-0.09	0.06	0.02	0.14	0.09	0.01	0.05	1		
H^+ plus Al^{3+}	-0.19	-0.27	-0.6***	0.82***	0.45***	0.03	0.21	0.12	1	
CTC	-0.18	-0.25	-0.59***	0.82***	0.47***	0.03	0.26	0.16	1***	1

***, $p \leq 0.001$; ** $p \leq 0.01$; * $p \leq 0.05$

Table S6.

Modified 27F oligonucleotide primer (5' -> 3')<br>Fusion *Ion torrent primer*, A, key signal, **barcode**, barcode adaptor, <u>27F primer</u>		Modified 338R oligonucleotide primer (3' -> 5')<br>Fusion *Ion torrent primer*, TrP1, <u>338R primer</u>	
27F 01	CCATCTCATCCCTGCGTGTCTCCGAC-TCAG-**CCTGAGATAC**-GAT-<u>AGAGTTTGATCMTGGCTCAG</u>	338R	CCTCTCTATGGGCAGTCGGTGAT-<u>TGCTGCCTCCCGTAGGAGT</u>
27F 02	CCATCTCATCCCTGCGTGTCTCCGAC-TCAG-**TTACAACCTC**-GAT-<u>AGAGTTTGATCMTGGCTCAG</u>	338R	CCTCTCTATGGGCAGTCGGTGAT-<u>TGCTGCCTCCCGTAGGAGT</u>
27F 03	CCATCTCATCCCTGCGTGTCTCCGAC-TCAG-**AACCATCCGC**-GAT-<u>AGAGTTTGATCMTGGCTCAG</u>	338R	CCTCTCTATGGGCAGTCGGTGAT-<u>TGCTGCCTCCCGTAGGAGT</u>
27F 04	CCATCTCATCCCTGCGTGTCTCCGAC-TCAG-**ATCCGGAATC**-GAT-<u>AGAGTTTGATCMTGGCTCAG</u>	338R	CCTCTCTATGGGCAGTCGGTGAT-<u>TGCTGCCTCCCGTAGGAGT</u>
27F 05	CCATCTCATCCCTGCGTGTCTCCGAC-TCAG-**TCGACCACTC**-GAT-<u>AGAGTTTGATCMTGGCTCAG</u>	338R	CCTCTCTATGGGCAGTCGGTGAT-<u>TGCTGCCTCCCGTAGGAGT</u>
27F 06	CCATCTCATCCCTGCGTGTCTCCGAC-TCAG-**CGAGGTTATC**-GAT-<u>AGAGTTTGATCMTGGCTCAG</u>	338R	CCTCTCTATGGGCAGTCGGTGAT-<u>TGCTGCCTCCCGTAGGAGT</u>
27F 07	CCATCTCATCCCTGCGTGTCTCCGAC-TCAG-**TCCAAGCTGC**-GAT-<u>AGAGTTTGATCMTGGCTCAG</u>	338R	CCTCTCTATGGGCAGTCGGTGAT-<u>TGCTGCCTCCCGTAGGAGT</u>
27F 08	CCATCTCATCCCTGCGTGTCTCCGAC-TCAG-**TCTTACACAC**-GAT-<u>AGAGTTTGATCMTGGCTCAG</u>	338R	CCTCTCTATGGGCAGTCGGTGAT-<u>TGCTGCCTCCCGTAGGAGT</u>
27F 09	CCATCTCATCCCTGCGTGTCTCCGAC-TCAG-**TTCTCATTGAAC**-GAT-<u>AGAGTTTGATCMTGGCTCAG</u>	338R	CCTCTCTATGGGCAGTCGGTGAT-<u>TGCTGCCTCCCGTAGGAGT</u>
27F 10	CCATCTCATCCCTGCGTGTCTCCGAC-TCAG-**TCGCATCGTTC**-GAT-<u>AGAGTTTGATCMTGGCTCAG</u>	338R	CCTCTCTATGGGCAGTCGGTGAT-<u>TGCTGCCTCCCGTAGGAGT</u>
27F 11	CCATCTCATCCCTGCGTGTCTCCGAC-TCAG-**TAAGCCATTGTC**-GAT-<u>AGAGTTTGATCMTGGCTCAG</u>	338R	CCTCTCTATGGGCAGTCGGTGAT-<u>TGCTGCCTCCCGTAGGAGT</u>
27F 12	CCATCTCATCCCTGCGTGTCTCCGAC-TCAG-**AAGGAATCGTC**-GAT-<u>AGAGTTTGATCMTGGCTCAG</u>	338R	CCTCTCTATGGGCAGTCGGTGAT-<u>TGCTGCCTCCCGTAGGAGT</u>
27F 13	CCATCTCATCCCTGCGTGTCTCCGAC-TCAG-**CTTGAGAATGTC**-GAT-<u>AGAGTTTGATCMTGGCTCAG</u>	338R	CCTCTCTATGGGCAGTCGGTGAT-<u>TGCTGCCTCCCGTAGGAGT</u>
27F 14	CCATCTCATCCCTGCGTGTCTCCGAC-TCAG-**TGGAGGACGGAC**-GAT-<u>AGAGTTTGATCMTGGCTCAG</u>	338R	CCTCTCTATGGGCAGTCGGTGAT-<u>TGCTGCCTCCCGTAGGAGT</u>
27F 15	CCATCTCATCCCTGCGTGTCTCCGAC-TCAG-**TAACAATCGGC**-GAT-<u>AGAGTTTGATCMTGGCTCAG</u>	338R	CCTCTCTATGGGCAGTCGGTGAT-<u>TGCTGCCTCCCGTAGGAGT</u>
27F 16	CCATCTCATCCCTGCGTGTCTCCGAC-TCAG-**CTGACATAATC**-GAT-<u>AGAGTTTGATCMTGGCTCAG</u>	338R	CCTCTCTATGGGCAGTCGGTGAT-<u>TGCTGCCTCCCGTAGGAGT</u>
27F 17	CCATCTCATCCCTGCGTGTCTCCGAC-TCAG-**TTCCACTTCGC**-GAT-<u>AGAGTTTGATCMTGGCTCAG</u>	338R	CCTCTCTATGGGCAGTCGGTGAT-<u>TGCTGCCTCCCGTAGGAGT</u>
27F 18	CCATCTCATCCCTGCGTGTCTCCGAC-TCAG-**AGCACGAATC**-GAT-<u>AGAGTTTGATCMTGGCTCAG</u>	338R	CCTCTCTATGGGCAGTCGGTGAT-<u>TGCTGCCTCCCGTAGGAGT</u>
27F 19	CCATCTCATCCCTGCGTGTCTCCGAC-TCAG-**CTTGACACCGC**-GAT-<u>AGAGTTTGATCMTGGCTCAG</u>	338R	CCTCTCTATGGGCAGTCGGTGAT-<u>TGCTGCCTCCCGTAGGAGT</u>
27F 20	CCATCTCATCCCTGCGTGTCTCCGAC-TCAG-**TTGGAGGCCAGC**-GAT-<u>AGAGTTTGATCMTGGCTCAG</u>	338R	CCTCTCTATGGGCAGTCGGTGAT-<u>TGCTGCCTCCCGTAGGAGT</u>
27F 21	CCATCTCATCCCTGCGTGTCTCCGAC-TCAG-**TGGAGCTTCCTC**-GAT-<u>AGAGTTTGATCMTGGCTCAG</u>	338R	CCTCTCTATGGGCAGTCGGTGAT-<u>TGCTGCCTCCCGTAGGAGT</u>
27F 22	CCATCTCATCCCTGCGTGTCTCCGAC-TCAG-**TCAGTCCGAAC**-GAT-<u>AGAGTTTGATCMTGGCTCAG</u>	338R	CCTCTCTATGGGCAGTCGGTGAT-<u>TGCTGCCTCCCGTAGGAGT</u>
27F 23	CCATCTCATCCCTGCGTGTCTCCGAC-TCAG-**TAAGGCAACCAC**-GAT-<u>AGAGTTTGATCMTGGCTCAG</u>	338R	CCTCTCTATGGGCAGTCGGTGAT-<u>TGCTGCCTCCCGTAGGAGT</u>
27F 24	CCATCTCATCCCTGCGTGTCTCCGAC-TCAG-**TTCTAAGAGAC**-GAT-<u>AGAGTTTGATCMTGGCTCAG</u>	338R	CCTCTCTATGGGCAGTCGGTGAT-<u>TGCTGCCTCCCGTAGGAGT</u>
